# Sex-Dependent Effects of Quadriceps Fat Content on Single Muscle Fiber Size in Older Adults

**DOI:** 10.1093/geroni/igaa057.419

**Published:** 2020-12-16

**Authors:** Chad Straight, Joseph Gordon, Aurora Foster, Nicholas Remillard, Bruce Damon, Stuart Chipkin, Jane Kent, Mark Miller

**Affiliations:** 1 University of Massachusetts Amherst, Amherst, Massachusetts, United States; 2 Vanderbilt University, Nashville, Tennessee, United States; 3 University of Massachusetts Amherst, Amherst, United States

## Abstract

Evidence suggests that ectopic fat deposition interferes with skeletal muscle structure and function, but few studies have examined underlying morphological and contractile properties at the single fiber level. Healthy older (65-75 y) men (n=9) and women (n=9) underwent dynamometry for assessment of knee extensor maximal torque, water-fat magnetic resonance imaging to quantify quadriceps muscle cross-sectional area (CSA) and fat fraction (FF), and vastus lateralis biopsies to determine morphology and function of type I and II muscle fibers. Despite similar body mass indices (24.4±1.3 vs. 24.6±0.5 kg∙m2, p=0.93) and daily moderate-to-vigorous physical activity (46±7 vs. 41±9 min∙d-1, p=0.67), women had greater FF (9.0±0.3 [range: 7.6-10.6] vs. 7.9±0.4 [6.0-9.7] %, p=0.04) than men, indicating increased adipose tissue deposition in skeletal muscle. Women also had smaller quadriceps CSA (39.8±1.8 vs. 57.9±1.3 cm2, p=0.01), specific torque (1.5±0.1 vs. 1.9±0.1 Nm∙cm-2, p=0.01) and type II fiber CSA (3,943±312 [2,350-5,140] vs. 5,352±384 [3,560-6,590] µm2, p=0.01) than men. Type I CSA did not differ by sex (4,918±228 [3,740-5,600] vs. 5,630±440 [3,640-7,670] µm2, p=0.19). In older women, FF was inversely associated with single fiber CSA in type I (r= -0.81, p=0.02) and II (r= -0.76, p=0.03) fibers, and tended to be associated with slower myosin-actin cross-bridge kinetics (longer myosin attachment time) in type I fibers (r=0.65, p=0.08). These relationships were not observed in men. Overall, healthy older women have greater intramuscular fat than men, which may contribute to sex-specific effects on knee extensor specific torque through differences in muscle fiber size and cross-bridge kinetics.

